# Upcycling of dynamic thiourea thermoset polymers by intrinsic chemical strengthening

**DOI:** 10.1038/s41467-022-28085-2

**Published:** 2022-01-19

**Authors:** Haijun Feng, Ning Zheng, Wenjun Peng, Chujun Ni, Huijie Song, Qian Zhao, Tao Xie

**Affiliations:** 1 State Key Laboratory of Chemical Engineering, College of Chemical and Biological Engineering, Zhejiang University, 38 Zheda Road, Hangzhou, 310027 P. R. China; 2 ZJU-Hangzhou Global Scientific and Technological Innovation Center, Hangzhou, 311215 P. R. China

**Keywords:** Mechanical properties, Polymers, Mechanical properties

## Abstract

Thermoset polymers are indispensable but their environmental impact has been an ever-increasing concern given their typical intractability. Although concepts enabling their reprocessing have been demonstrated, their practical potential is limited by the deteriorated performance of the reprocessed materials. Here, we report a thiourea based thermoset elastomer that can be reprocessed with enhanced mechanical properties. We reveal that the thiourea bonds are dynamic which leads to the reprocessibility. More importantly, they can undergo selective oxidation during high temperature reprocessing, resulting in significant chemical strengthening within certain reprocessing cycles. This is opposite to most polymers for which reprocessing typically results in material deterioration. The possibility of having materials with inherent reprocessing induced performance enhancement points to a promising direction towards polymer recycling.

## Abstract (web summary)

The ability to reprocess thermoset polymers has been demonstrated in principle, but is not widely done due to deteriorated performance of the reprocessed materials. Here, we report a thiourea-based thermoset elastomer that can be reprocessed with enhanced mechanical properties.

## Introduction

The discovery of synthetic polymers has brought enormous benefits to the human society. However, their excessive use has led to concern on the negative impact to the environment^1^. Thermoplastic polymers can be reprocessed^2^, but their performance is generally reduced, limiting their practical value. By comparison, classical thermosets are not reprocessible due to their permanent cross-linked nature. Despite such, thermosets remain indispensable given their unique advantages (e.g., dimension stability) over thermoplastic polymers^3^. Thermoset rubbers in particular have been widely used in many aspects of our daily lives including tires, sealants, adhesives, and medical devices. To mitigate the negative environmental effect of thermosets, extensive efforts have been made to develop new technologies to recycle them^4–8^. A particularly attractive approach is to make them reprocessible by introducing dynamic covalent bonds into the networks^9–21^ or activating the dynamic characteristics of the otherwise conventional covalent bonds with catalysts^22–25^. Despite the promising developments at the experimental scale, successful implementation at the commercial level has been quite limited (if any). One major reason is that reprocessed thermosets typically experience unavoidable mechanical performance deterioration. During reprocessing, three destructive molecular events generally happen: chain scission, degradation, and oxidation. The first one is due to the encountering of large mechanical forces (e.g., grinding) whereas the latter two originate from the exposure to high temperature under realistic conditions in which oxygen/water is present. In principle, better mechanical recovery is possible if side reactions could be alleviated. Indeed, for a thioureathane network, introducing excess thiol groups was shown to effectively counter the side reaction between isocyanates and environmental water, leading to full mechanical recovery even after three reprocessing cycles^24^. However, the above approach does not allow upcycling for which the reprocessed materials can show better mechanical properties than the original material.

In this work, we hypothesize that a more effective approach is to develop a proactive mechanism that can counteract all the side effects that lead to mechanical deterioration. Such an approach potentially can even make the reprocessed materials stronger than the original ones. Hereafter, we describe our successful attempt in this direction with a dynamic thiourea-based thermoset elastomer. Our original motivation of exploring thiourea bonds is two folds: thiourethane bonds are more readily dynamic than urethane bonds^24^; urea and hindered urea bonds are both dynamic with the steric hindrance in the latter enhancing the dynamic exchangeability^26^. We emphasize that non-cross-linked thiourea polymers are known to exhibit unusual room temperature self-healing behavior via their unique zig-zag hydrogen bonds^27^, but such a principle is different from the utilization of the dynamic covalent characteristics for designing reprocessable thermosets.

## Results

### Polymer network design and the principle for strengthening

**Fig. 1:**
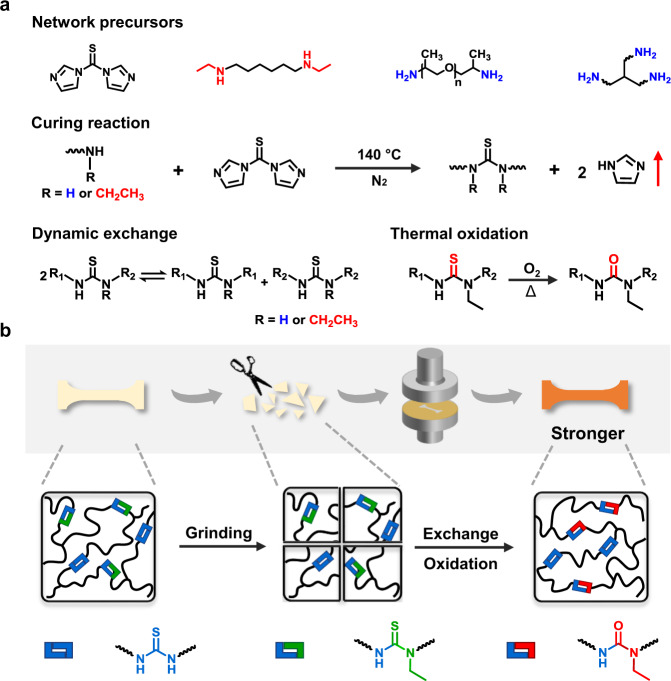
Polymer network design and the principle for strengthening. **a** The chemistry for network synthesis, reprocessing, and oxidation. **b** Schematic illustration of reprocessing and strengthening of the cross-linked network.

The enabling chemistry is shown in Fig. 1a. The network is synthesized by reacting thiocarbonyldiimidazole with a primary diamine and a secondary diamine, along with a triamine cross-linker. The curing reaction proceeds at 140 °C under nitrogen flow without any catalyst, through a substitution reaction^27^ with the byproduct imidazole removed by the gas flow. Two types of thiourea bonds are produced, namely, a non-hindered thiourea from the primary amine and a hindered thiourea from the secondary amine. We note here that linear polymers containing thiourea bonds have been reported in the literature^27,28^, but their dynamic covalent nature is not known. Our discovery here is that the thiourea bonds are dynamic covalent bonds. The other discovery is that the hindered thiourea bonds can undergo controllable thermal oxidation in air to yield the corresponding urea. As illustrated in Fig. 1b, the dynamic thiourea exchange reaction enables the reprocessing of the cross-linked polymer. Surprisingly, the conversion from the hindered thiourea to the urea as the result of the otherwise undesirable thermal oxidation yields a strengthening effect, making the reprocessed polymer stronger than the original one.

### Dynamic property of thiourea network

**Fig. 2:**
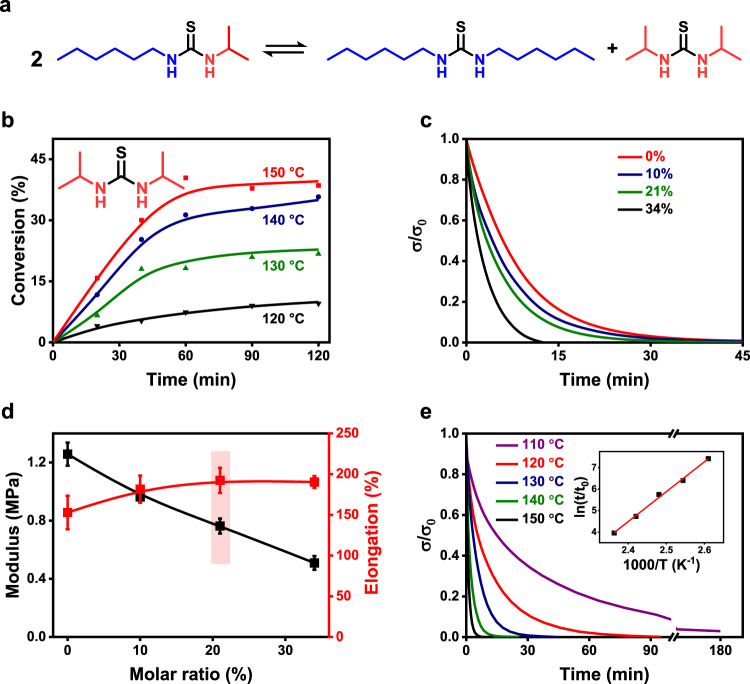
Dynamic property of thiourea network. **a** The exchange reaction of the model compound. **b** Exchange kinetics of the model compound. **c** Isostrain stress-relaxation curves of polymer networks containing different amounts of the hindered thiourea bond (strain: 20%; temperature: 130 °C). **d** Mechanical properties of polymer networks with different amounts of the hindered thiourea bond. **e** Isostrain stress-relaxation curves of the polymer network with 21% of the hindered thiourea bond at different relaxation temperatures (strain: 20%).

The dynamic nature of the thiourea bond is verified through an exchange reaction conducted with *1*-hexyl-*3*-isopropyl-thiourea as the small molecular model compound. Its self-exchange reaction is expected to yield *1,3*-diisopropylthiourea and *1,3*-dihexylthiourea (Fig. 2a), which is confirmed by gas chromatographic analyses presented in Supplementary Fig. 1. Detecting *1,3*-diisopropylthiourea by gas chromatography therefore allows monitoring the exchange reaction. Accordingly, Fig. 2a shows that heating promotes the exchange reaction until reaching the equilibrium states at about one hour between 130 and 150 °C. The activation energy for the thiourea exchange is determined as 69.1 kJ/mol (Supplementary Fig. 1).

Having verified the dynamic thiourea exchangeability with the small molecule, we proceed to investigate their impact on polymer networks. A series of cross-linked polymers (detailed formulations in Supplementary Table 1) are synthesized with an identical cross-linking density but variation in the molar percent of the hindered thiourea with respect to the total thioureas. These samples are subjected to isostrain stress relaxation experiments at 130 °C. The results (Fig. 2c) show that all of them exhibit complete stress relaxation. The stress relaxation is faster for samples with higher amounts of the hindered thiourea, suggesting that the steric hindrance promotes faster exchange, similar to the trend known for urea bonds^28–30^.

**Fig. 3:**
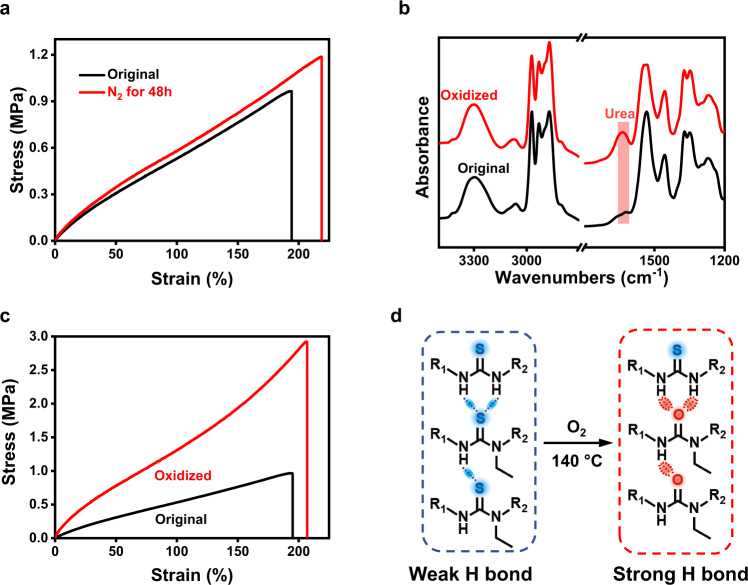
Selective oxidation of the hindered thiourea bond. **a** The mechanical property of samples before and after thermal treatment (140 °C, 48 h) in nitrogen flow. **b** Infrared spectra of the thiourea network (without reprocessing) before and after thermal oxidization in air for 48 h at 140 °C. **c** Thermal oxidation (in air) induced mechanical property change of the thiourea network (without reprocessing). **d** Formation of different hydrogen bonding due to the oxidation.

The hindered thiourea bond in the network affects the mechanical properties of the materials. As its molar ratio increases from 0 to 34% (Fig. 2d), the modulus decreases linearly between 1.25 and 0.51 MPa. By comparison, its elongation at break raises gradually within a relatively narrow range between 152 and 190%. Unless otherwise noted, we hereafter choose the polymer sample with 21% hindered thiourea bonds (light red rectangle in Fig. 2d) for further investigation. The sample does not dissolve but swells in a range of solvents (gel content: 83 ± 5%, see Supplementary Table 2), confirming its cross-linked nature. In addition, the sample is also chemically stable against acidic and basic *N,N*-dimethylformamide. Nevertheless, Fig. 2e illustrates that this sample can undergo faster stress relaxation at higher temperatures with a corresponding activation energy of 115.8 kJ/mol calculated from the inset figure. Complete stress relaxation is reached at a relatively short time of 15–20 min at 140 °C, which is chosen as the appropriate reprocessing temperature. We emphasize here that the sample is a fully reacted network since further thermal post-treatment under nitrogen does not result in any change to the material, including the composition, mechanical and thermal properties (Supplementary Fig. 2 and Fig. 3a). This is a critical reference for the thermal oxidation enhanced mechanical properties presented later in the context.

### Selective oxidation of the hindered thiourea bond

As illustrated in Fig. 1, the most critical concept here is that the dynamic network can not only be reprocessed, but also undergo constructive thermal oxidation to enhance the mechanical properties. To verify the thermal oxidation reaction, a hindered thiourea (Supplementary Fig. 3) is chosen as a small molecular model compound. It is exposed to an oxygen atmosphere at 140 °C. ^1^H-NMR (Supplementary Fig. 3) of the oxidized products in comparison with the original compound suggest that hindered urea is indeed progressively produced. HPLC-MS analysis (Supplementary Fig. 4) further confirms that the oxidation converts the hindered thiourea into the corresponding urea, along with some side products corresponding to a molecular weight increment of 14 (the molecular weight of methylene) from these two compounds. We note here that HPLC-MS is only qualitative since the sensitivity of each compound is unknown. Nevertheless, the ^1^H NMR (Supplementary Fig. 3) suggests that these side products are minor compared to the main oxidation product (i.e., urea). In the infrared analyses (Fig. 3b), the appearance of a urea characteristic band at 1640 cm^−1^ after oxidation further verify the oxidation reaction. By comparison, an unhindered thiourea is found to be stable under the same oxidation conditions (Supplementary Fig. 5). From the mechanism standpoint, the conversion from thiourea to urea follows two steps: (1) oxygen forms an intermediate complex with the thiourea; (2) the complex loses sulfur monoxide to form urea^31^.

The oxidation-induced conversion from the hindered thiourea to the urea is found to significantly enhance the mechanical performance of the network polymer. The stress-strain curves of the synthesized sample before and after oxidation for 48 h in air at 140 °C (without reprocessing) are presented in Fig. 3c. The original material has a modulus of 0.74 MPa, strength of 0.96 MPa, respectively. After the oxidation, the modulus and strength are markedly increased to 2.06 and 2.88 MPa, while the thermal treatment in nitrogen hardly make any differences in mechanical properties (Fig. 3a). Notably, even the maximum strain is raised from 192 to 210% after oxidation. The strengthening of the all-around mechanical performances is surprising as common knowledge suggests that polymer oxidation usually leads to deterioration of mechanical properties (e.g., becoming more brittle). We believe such a desirable effect arises from the ability of thiourea and urea to form hydrogen bonds of different strengths. Compared with the sulfur atom, the oxygen atom in urea has higher electronegativity and smaller atomic radius, with both attributes favoring stronger hydrogen bonds^32^. The existence of hydrogen bonds in both the original and reprocessed samples (with 24 h of oxidation) is verified by the temperature ramping FTIR analysis. The results (Supplementary Fig. 6) show the weakening of hydrogen bonding (3299 cm^−1^) upon heating accompanied by the appearance of free N–H bond (3420 cm^−1^) and such a trend is reversed upon cooling. We note that, while the network polymer can be oxidized at elevated temperatures, it is stable under ambient condition. Its mechanical properties remain largely unchanged after being kept in ambient air for 60 days (Supplementary Fig. 7).

### Network reprocessing and enhancement in mechanical properties

**Fig. 4:**
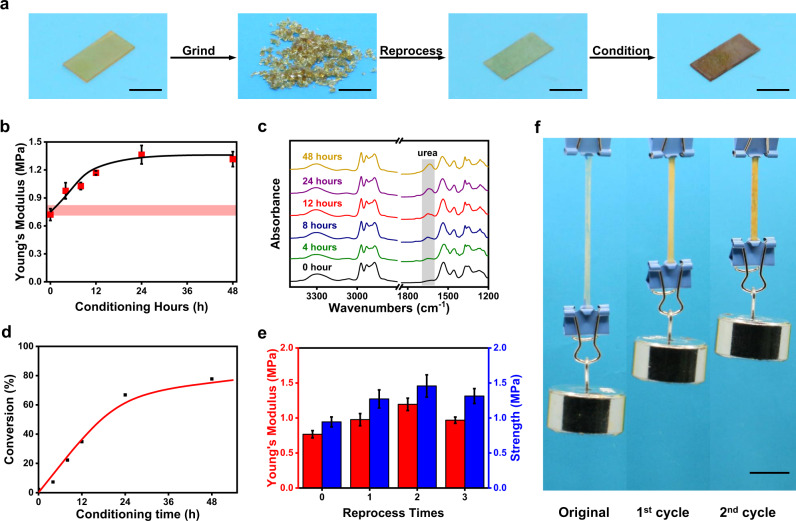
Network reprocessing and enhancement in mechanical properties. **a** Photographic images of the sample at different stages during the reprocessing. **b** Change in Young’s modulus of the reprocessed samples with different conditioning time in air. **c** FTIR change of the reprocessed samples with different conditioning time (in air). **d** Hindered thiourea conversion of the reprocessed samples with different conditioning time. **e** Change in Young’s modulus and strength at different reprocessing cycles. For each reprocessing cycle, the conditioning time is 4 h. **f** Photographic images of the thiourea sample with different reprocessing cycles under a constant load of 50 g. The sample size without the external load is 15 × 3 × 0.5 mm. All scale bars are 1 cm.

The dynamic characteristics of the thiourea bonds, along with the favorable oxidation chemistry, establishes an unusual basis for a cross-link polymer to be reprocessed into a stronger material. For reprocessing, the polymer is ground into small pieces and mold-pressed (4 MPa, 30 min, 140 °C) (Fig. 4a). To harness the benefit of the oxidation, an important conditioning step (140 °C in open air) is performed after the pressure molding.

The impact of this chemical change on the mechanical properties of the reprocessed polymer are demonstrated in Fig. 4b and Supplementary Fig. 8. Without conditioning (conditioning time of 0), the reprocessed material has a modulus lower than that of the original material highlighted as the light red rectangle. Conditioning for around 0.5 to 1 h would recover the modulus value. Further conditioning results in significant modulus increase until reaching a plateau value at around 18 h. The enhancement of the strength follows a similar trend although the time for reaching plateau is shorter (around 12 h). The plateau observed in Fig. 4b is mainly due to the fact the destructive thermal degradation is offset by the constructive oxidation. Upon further oxidation for 72 h, the material does become noticeably more brittle. In accordance with the mechanical change, the peak corresponding to urea (1640 cm^−1^) is progressively enhanced in the FTIR spectra during the conditioning step in air (Fig. 4c). To quantify the hindered thiourea to urea conversion, a network sample is synthesized with its urea and thiourea contents equal to that of the above network assuming that all the hindered thiourea are converted into urea in the conditioning step. This sample is used as a reference for FTIR analysis of the reprocessed and conditioned samples (Supplementary Fig. 9). The results show that the conversion from the hindered thiourea to urea upon conditioning (Fig. 4d) follows a similar trend with the mechanical property (Fig. 4b, Supplementary Fig. 8), verifying the molecular mechanism.

To highlight the uniqueness of the oxidation enhanced strengthening, a polyurea sample with a similar network structure is subjected to reprocessing at 180 °C, followed by oxidation in air at 140 °C for 4 h. As expected, the mechanical performance of the polyurea deteriorates markedly after reprocessing (Supplementary Fig. 10). The oxidation-induced strengthening is effective after repeated reprocessing. The same polythiourea sample can undergo multiple reprocessing cycles with a conditioning time of 4 h. Figure 4e shows that both the modulus and strength are progressively increased in the first two reprocessing cycles. The photographic images in Fig. 4f provide visual evidence of the modulus enhancement in the first two cycles. After the third reprocessing cycle, the mechanical performance is declined, but still higher than that of the original sample.

From a practical standpoint, a reprocessed material only has to maintain the original mechanical properties in order to meet the requirement for its reuse. Therefore, the conditioning time can be shortened and the reprocessing cycles can exceed three. On the other hand, if upcycling is the goal, the enhancement in the mechanical properties can be finely tuned by adjusting the conditioning time. Indeed, upcycling beyond two cycles is possible by shortening the conditioning time in each cycle. This is experimentally verified with the data presented in Supplementary Fig. 11, showing four reprocessed cycles with notable mechanical improvement. We note that, the conversion of hindered thiourea into urea is accompanied with a weight loss due to the removal of the sulfur. This maximum weight loss is, however, very small (around 1.6 wt%), assuming all the hindered thiourea turned into urea.

In addition, scaling up the above material is possible, but the curing time and synthesis conditions are the economical and practical factors that should be considered. The side products including imidazole generated in the synthesis and gas generated during oxidation should be collected to avoid any negative environmental impact. An alternative approach to taking advantage of the material chemistry principle is to synthesize oligomers that contain the active species (thiourea bonds) and use them as starting materials for making other types of thermosets with a more conventional curing chemistry. This is a direction we are going in the future.

## Discussion

Deterioration of mechanical properties after reprocessing is a major bottleneck for the sustainable uses of polymers. During reprocessing, a range of non-clean side reactions collectively contribute to the performance drop. While some of them can be mitigated (e.g., oxidation), the real challenge is to contain all of major ones given their random nature. This work reveals the dynamic characteristics of thiourea bonds, which enables the reprocessing of the network. More importantly, it utilizes the unique oxidation chemistry of hindered thiourea bonds as a mechanism for compensating the performance drop. This strategy is effective regardless of what side reactions may happen during the reprocessing. Although the upcycling is not unlimited, the materials embedded with thiourea bonds do achieve mechanical enhancement within certain reprocessing cycles. The intrinsic strengthening effect can be potentially extended beyond the thiourea chemistry. The work overall points to a promising direction in designing reprocessable polymers with markedly improved lifetime.

## Methods

### Materials

The chemicals used were thiocarbonyldiimidazole (TCDI, Aladdin, 95%), poly (propylene glycol) bis (2-aminopropyl ether) (D400, Sigma Aldrich, Mn~400), trimethylolpropane tris [poly (propylene glycol), amine terminated] ether (T440, Sigma Aldrich, Mn~440), *N,N*′-diethyl-1,6-diaminohexane (DDAH, TCI, 97%), *N,N*′-carbonyldiimidazole (Aladdin, 97%), isopropyl isothiocyanate (TCI, 98%), *N*-ethylbutylamine (TCI, 99%), *N,N*′-diisopropylthiourea (TCI, 99%), hexylamine (TCI, 99%), isopropyl isocyanate (TCI, 98%), di-tert-butylbipheny (TCI, 98%), benzyl isothiocyanate (TCI, 98%), dimethylformamide (Sinopharm, 99%), tetrahydrofuran (Sinopharm, 99%), dichloromethane (Sinopharm, 99%), toluene (Sinopharm, 99%), ethyl acetate (Sinopharm, 99%), acetone (Sinopharm, 99%), ethanol (Sinopharm, 99%), ether (Sinopharm, 99%), hexane (Sinopharm, 99%), sodium hydroxide (Sinopharm, 99%), hydrochloric acid (Sinopharm, 38% in water). All chemicals were used as received.

### Synthesis of the thiourea networks

Based on the formulation provided in Supplementary Table 1, D400 and DDAH were dissolved in 2 mL of dimethylformamide. T440 and TCDI were introduced afterward. The mixture was stirred at room temperature until thiocarbonyldiimidazole was fully dissolved. The transparent solution was then poured into a Teflon mold and thermally cured at 80 °C for 6 h under nitrogen flow. The sample was post-cured at 140 °C for 12 h under nitrogen flow, yielding a yellow transparent thin film (with the thickness controlled at about 0.5 mm). The imidazole byproduct was removed by the nitrogen flow and then entered to a cooling equipment. During the cooling process, imidazole crystallized into solid for easy recovery.

### Thiourea network reprocessing

In a typical experiment, the polymer film was cut into small pieces, put into a 0.5-mm-thick iron mold, and pressed for 30 min at 140 °C under 4 MPa. The reformed polymer film was taken out the mold and kept in an oven (air environment) at 140 °C for conditioning to yield a reprocessed sample. The above process was repeated for a subsequent reprocessing cycle.

### Isostrain stress relaxation experiments

All the tests were performed using a DMA Q800 (TA instruments) in the “stress relaxation” model. In a typical test, the thin film sample was heated to the target temperature and stretched to 20% strain. The stress in the sample was monitored and recorded while the strain was maintained constant.

### Tensile test

The samples used for testing were cut into rectangular shapes (10 × 3 × 0.5 mm). All tensile tests were carried out using a Zwick/Roell Z005 tensile machine. The experiments were conducted at 25 °C at a strain rate of 10 mm/min.

### Other analyses

Gas chromatography analyses were carried out using an Agilent 6890 machine using di-tert-butylbipheny as the internal standard and tetrahydrofuran as the solvent. Infrared spectrum (FTIR) analyses were conducted with thin film samples using Nicolet5700 in attenuated total reflection (ATR) mode. Temperature ramping FTIR analyses were conducted in transmission mode. HPLC-MS analyses were performed using Agilent 6545 with THF as the solvent. All the ^1^H-NMR analyses were conducted in CDCl_3_ with a Bruker AVANCE III (500 MHz) machine.

## Supplementary information

Supplementary information

## Data Availability

All data that support the findings of this study are available from the corresponding author on reasonable request.
