# Supplementary material for: Upcycling of dynamic thiourea thermoset polymers by intrinsic chemical strengthening
Source: Nat Commun. 2022 Jan 19;13:397. doi: 10.1038/s41467-022-28085-2 (PMC8770626; doi:10.1038/s41467-022-28085-2)
Supplement: Supplementary file 1 — Supplementary information [file 41467_2022_28085_MOESM1_ESM.pdf]

Supplementary Materials for

Upcycling of dynamic thiourea thermoset polymers by intrinsic chemical strengthening

Haijun Feng[1,2], Ning Zheng[1*], Wenjun Peng[1], Chujun Ni[1], Huijie Song[1], Qian Zhao[1,2], Tao Xie[1,2]

Correspondence to: zhengning@zju.edu.cn

Supplementary Methods

Residual imidazole analysis. The residual imidazole in the synthesized thiourea networks was estimated by the Gas Chromatography (GC) analysis. First, 118 mg of the synthesized films was fully swelled in 1.5 mL of THF. The swelling solution was then collected and analyzed by GC, from which the concentration of imidazole in THF was calculated through an internal standard (di-tert-butylbipheny, 2.5 mg/mL). Based on this analysis, the content of the imidazole was 2.5 wt%.

Synthesis and reprocessing of polyurea network: 1.0 g of D400 was dissolved in 2 mL of dimethylformamide. 1.0 g of T440 and 0.96 g of *N,N'*-carbonyldiimidazole were introduced afterwards. The mixture was stirred at room temperature until *N,N'*-carbonyldiimidazole was fully dissolved. The transparent solution was then poured into a Teflon mold and cured at 80 °C for 6 hours under nitrogen flow. The sample was post-cured at 140 °C for 12 hours under nitrogen flow to yield a transparent film. Reprocessing of the polyurea film follows the same process for thiourea polymer except that the molding temperature was 180 °C and the condition time was 4 hours.

Synthesis of a reference network polymer for FTIR analysis: 0.2 g of DDAH and 0.38 g N,N'-carbonyldiimidazole (molar ratio: 1:2) were dissolved in 1 g DMF. The mixture was reacted at room temperature for 4 hours. Afterwards, 0.8 g of D400, 1.0 g of T400 and 1.171 g TCDI (the molar ratio of amine group to carbonyl/thiocarbonyl group is 2:1) were introduced to the mixture. After TCDI was fully dissolved, the mixture was poured into a Teflon mold, and reacted at 80 °C under nitrogen flow for 6 hours. Afterwards, the temperature was adjusted to 140 °C for another 12 hours reaction. The urea and thiourea contents in this reference polymer are equal to that of the network with 21% hindered thiourea bonds, if all the hindered thiourea are turned into urea in the conditioning step.

Quantification of thiourea conversion during conditioning: The conversion (*C*) of the hindered thiourea for the reprocessed samples was analyzed by comparing their FTIR spectra with the reference's, using the following equation:

$$C = \frac{A_{u,c} \cdot A_{CH,r}}{A_{CH,c} \cdot A_{u,r}} \times 100\%,$$

where $A_{u,c}$, $A_{CH,c}$, $A_{u,r}$ $A_{CH,r}$ are the respective peak areas of the urea band of the conditioned samples (1640 cm^{-1}), C-H band of the conditioned samples (2810-3020 cm^{-1}), urea band of the reference sample (1640 cm^{-1}), and C-H band of the reference sample (2810-3020 cm^{-1}).

Model compound bond exchange experiments: The model compound *1*-hexyl-*3*-isopropyl-thiourea (HITU) was synthesized by the reaction of isopropyl isothiocyanate and hexylamine. 1.00 g of isopropyl isothiocyanate and 1.00 g of hexylamine were added into a glass bottle (the molar ratio of thiocyanate and amine is 1:1). After being stirred for 12 hours at room temperature, white powders were obtained, which were vacuum dried for 24 h to yield the final product. Its chemical structure was verified by ^{1}H-NMR analysis (Supplementary Fig. 12).

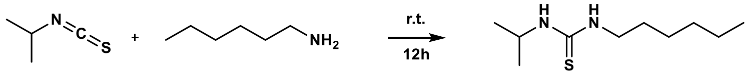

For bond exchange experiments, a series of 50 mg HITU were added into different glass bottles and heated to desired temperatures (120 °C-150 °C). Samples were taken out of the bottles at different times (20 min, 40 min, 60 min, 90 min, and 120 min) for gas chromatography analyses.

Model compound oxidation experiments: The model compound *1*-butyl-*1*-ethyl-*3*-isopropyl-thiourea (BEIT) was synthesized by the reaction of isopropyl isothiocyanate and *N*-ethylbutylamine. 1.00 g isopropyl isothiocyanate and 1.00 g of *N*-ethylbutylamine were added into a glass bottle (the molar ratio between thiocyanate and amine is 1:1). After being stirred for 12 hours at room temperature, a yellow liquid was obtained, which was vacuum dried for 24 hours to yield the final product. Its chemical structure was verified by ^{1}H-NMR analysis (Supplementary Fig. 13).

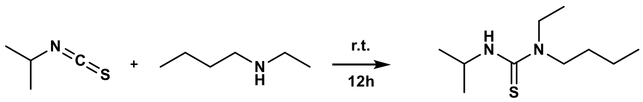

For the oxidation experiments, 0.40g of BEIT was heated to 140 °C in a glass bottle under oxygen atmosphere. At reaction times of 12 hours and 24 hours, about 0.10 g of the reaction product was collected for ^{1}H-NMR analysis.

For comparison, a non-hindered *N,N'*-diisopropylthiourea was also oxidized under the same condition and the product was collected after reaction for 24 hours for ^{1}H-NMR analysis.

Synthesis of 1-butyl-1-ethyl-3-isopropyl-urea: 1-butyl-*1*-ethyl-*3*-isopropyl-urea was synthesized as a reference for ^{1}H-NMR analysis of the oxidized product of BEIT. The reaction between isopropyl isocyanate and *N*-ethylbutylamine proceeded in an identical manner as BEIT and the molar ratio between cyanate and amine was 1:1.

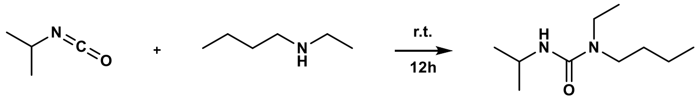

Swelling tests: Weighted thiourea polymers (approximately 35 mg) were soaked in 10 mL of different solvents at room temperature until reaching the swelling equilibrium. The samples were subsequently dried under vacuum until reaching a constant value. The gel contents were calculated as: 100%×final weight/initial weight.

Supplementary Figures

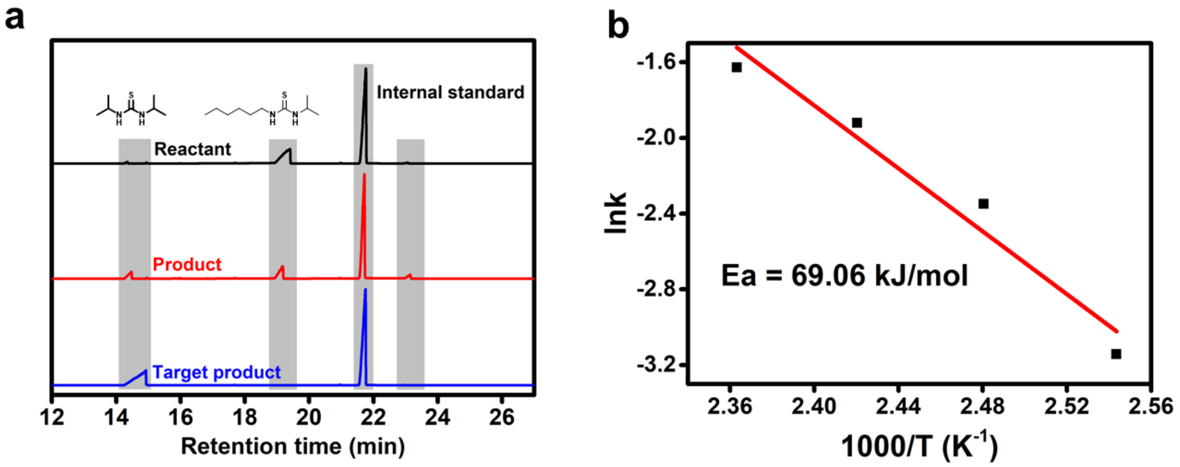

Supplementary Fig. 1 | a. Representative gas chromatography spectra showing that the two new peaks appeared after the bond exchange. The peak intensity of *N*,*N'*-diisopropylthiourea was used to monitor the exchange kinetics. **b.** The Arrhenius curve for the self-exchange reaction of *1*-hexyl-*3*-isopropyl-thiourea, obtained from the gas chromatographic analyses.

Supplementary Table 1 | Formulations of the thiourea networks

Molar percent	DDAH (10^{-3} mol)	D400 (10^{-3} mol)	T440 (10^{-3} mol)	TCDI (10^{-3} mol)
0%	0.00	2.50	2.27	5.91
10%	0.58	2.25	2.27	6.23
21%	1.16	2.00	2.27	6.57
34%	1.74	1.75	2.27	6.90

Supplementary Table 2 | Solvent swelling and resistance of the thiourea network

Solvent	Swelling ratio (%)	Gel content (%)
N,N-Dimethylformamide	466	85.4
Tetrahydrofuran	422	78.2
Dichloromethane	547	78.6
Toluene	153	88.2
Ethyl acetate	180	82.4
Acetone	226	80.8
Ethanol	235	83.7
Ether	115	88.0
Hexane	Non-swelling	
Acidic DMF [a]	464	87.3
Basic DMF [b]	236	88.0

[a]: the acidic solvent was obtained by mixing 3.65 g HCl water solution in DMF (1 L). [b]: the basic solvent was obtained by dissolving 4.00 g NaOH in a mixture of water (0.1 L) and DMF (0.9 L).

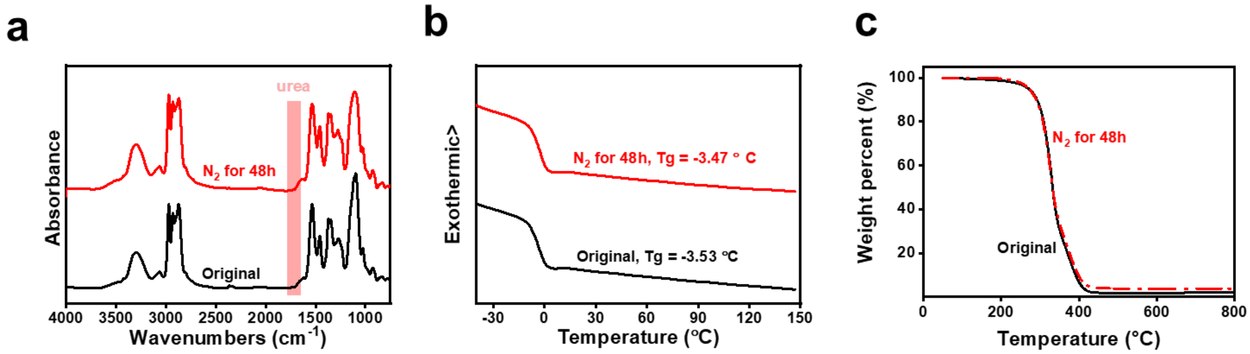

Supplementary Fig. 2 | Comparison of the infrared spectra (a), DSC curves (b) and TGA curves (c) of the as synthesized and post-treated thiourea networks. The post-treatment was conducted at 140 °C for 48 hours under nitrogen flow. None of the infrared spectrum, the DSC curves and TGA curves show any notable changes after the post-treatment, suggesting that the original as synthesized network was fully cured.

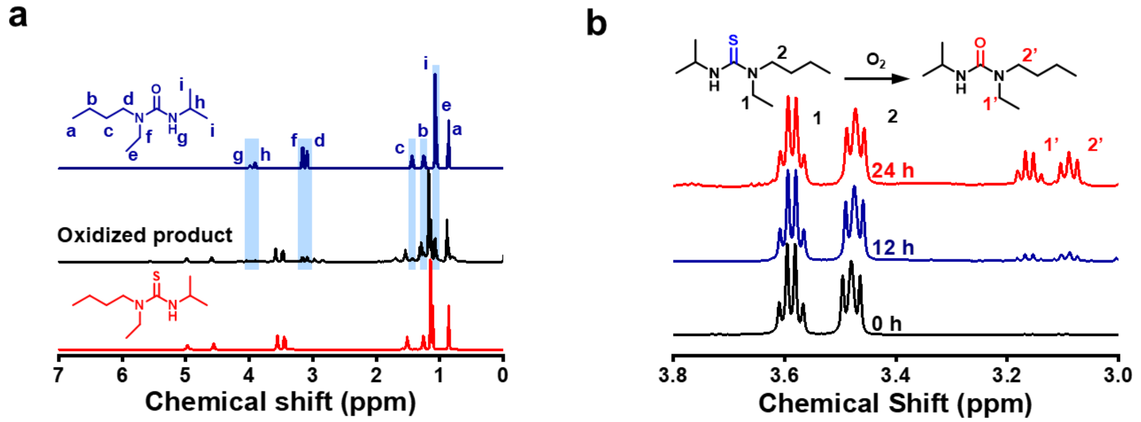

Supplementary Fig. 3 | ^{1}H-NMR analysis of the oxidization behavior of *1*-butyl-*1*-ethyl-*3*-isopropyl-thiourea (BEIT). a, Full ^{1}H-NMR spectra of the original thiourea, oxidized products (24 hours in oxygen, 140 °C) and resulting urea. The comparison among these three spectra suggests the thiourea to urea is the main reaction. **b,** Time evolution of ^{1}H-NMR spectra of BEIT upon oxidation in oxygen. We emphasize here that, some unknown minor side products are also present, which highlights the complexity of oxidation reaction and the challenge for polymer reprocessing. On the flip side, it proves the effectiveness of our overall approach in countering side reactions with a constructive reaction.

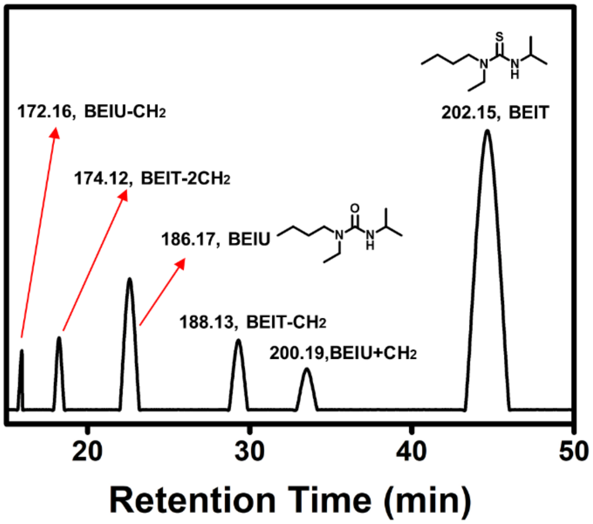

Supplementary Fig. 4 | HPLC-MS analysis of the oxidized product of 1-butyl-1-ethyl-3-isopropyl-thiourea (BEIT). Each peak is labeled with its main m/z number. The starting 1-butyl-1-ethyl-3-isopropyl-thiourea and its corresponding oxidized urea (BEIU) are detected. In addition, side products corresponding to molecular weight increments of 14 from these two compounds are also identified.

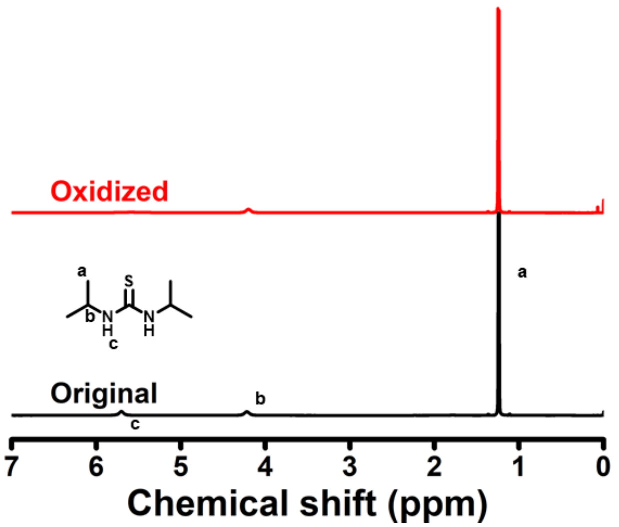

Supplementary Fig. 5 | ^{1}H-NMR spectra of *N*,*N'*-diisopropylthiourea before and after oxidation.

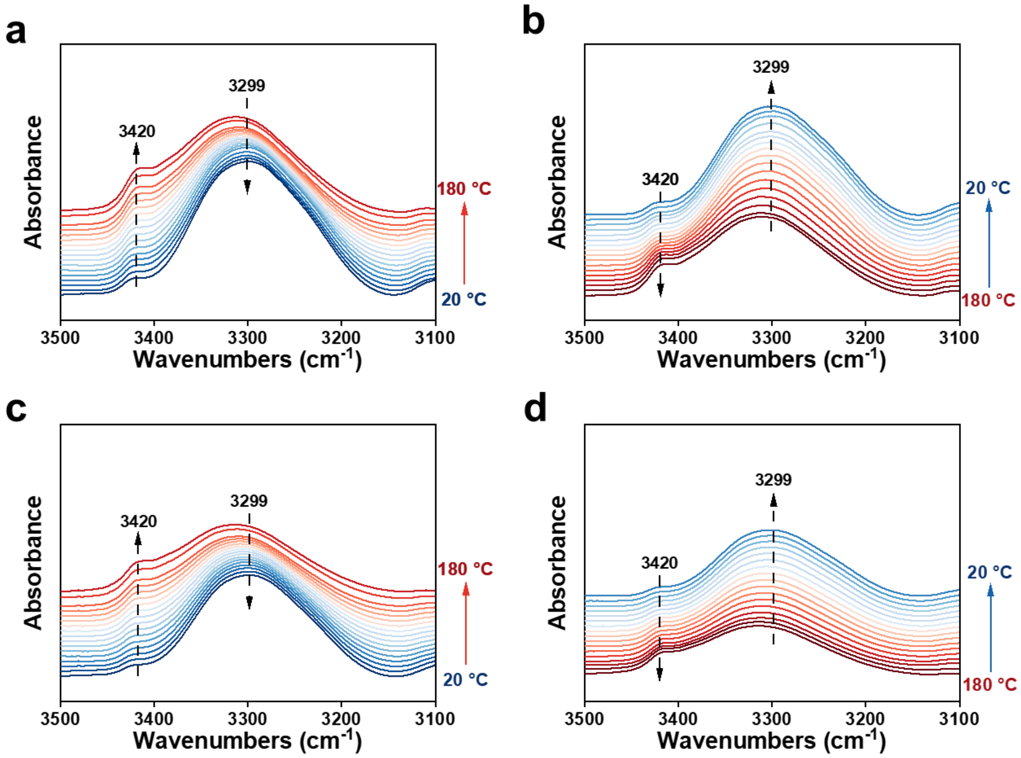

Supplementary Fig. 6 | Temperature ramping FTIR spectra of the original (a, heating; b, cooling) and reprocessed (c, heating; d, cooling) samples. The conditioning time (in air) of the reprocessed sample is 24 hours.

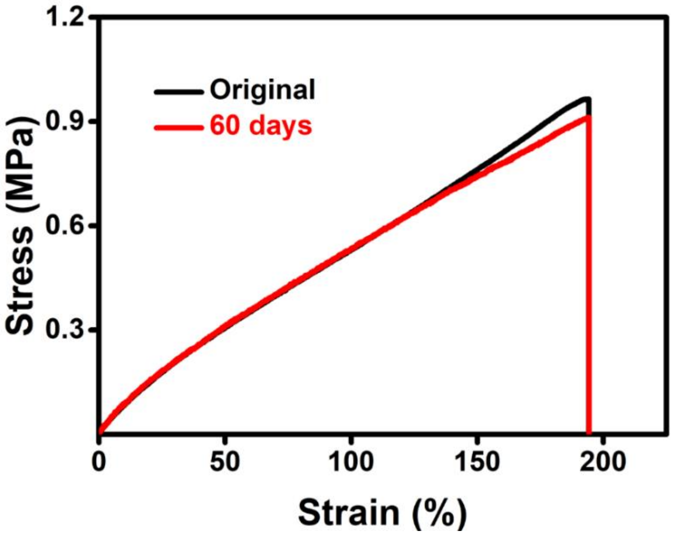

Supplementary Fig. 7 | Mechanical curve of polythiourea before and after being kept under ambient condition for 60 days.

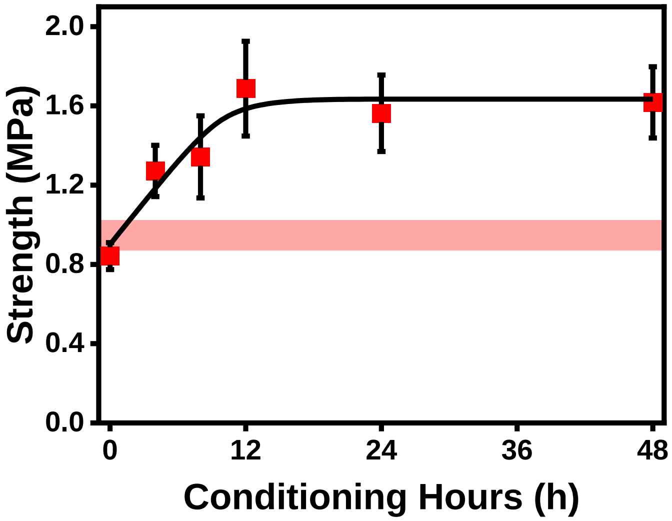

Supplementary Fig. 8 | Change in strength upon conditioning of the reprocessed samples. The light red rectangle refers to the strength of the original sample.

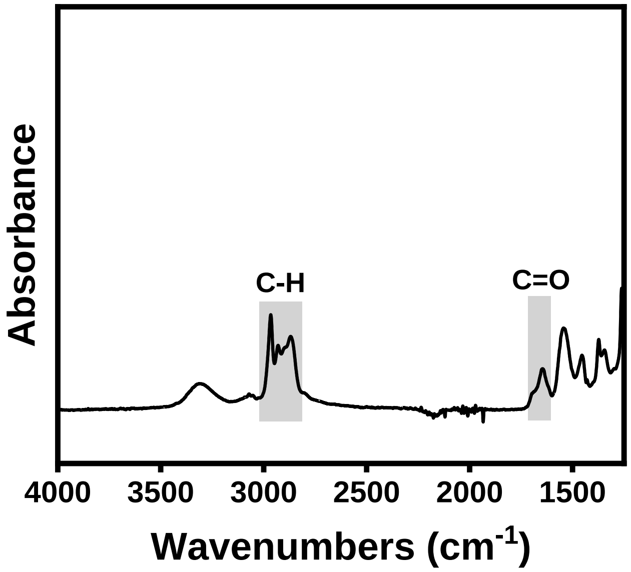

Supplementary Fig. 9 | FTIR of the reference sample

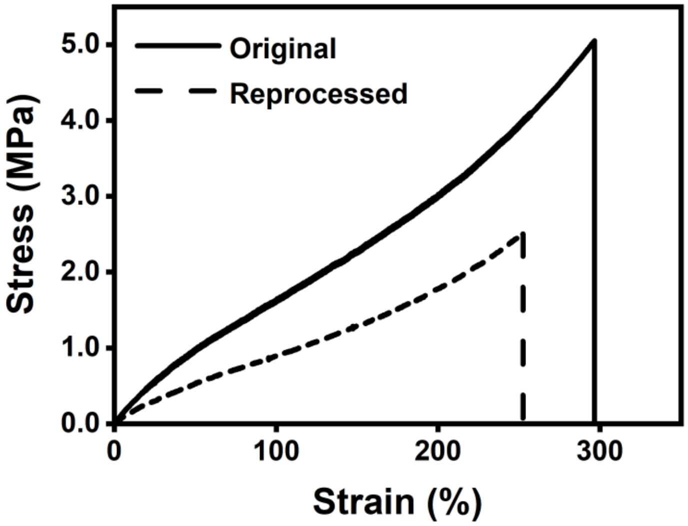

Supplementary Fig. 10 | Stress-strain curves of a comparative polyurea before and after reprocessing in air, with thermal conditioning time of 4 h.

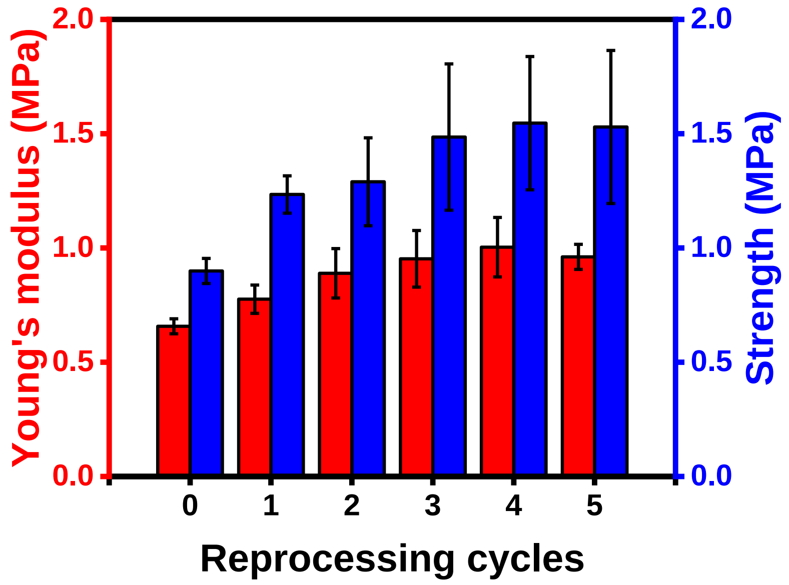

Supplementary Fig. 11 | The mechanical properties of the reprocessed samples upon repeated recycling with 2 hours conditioning for each cycle.

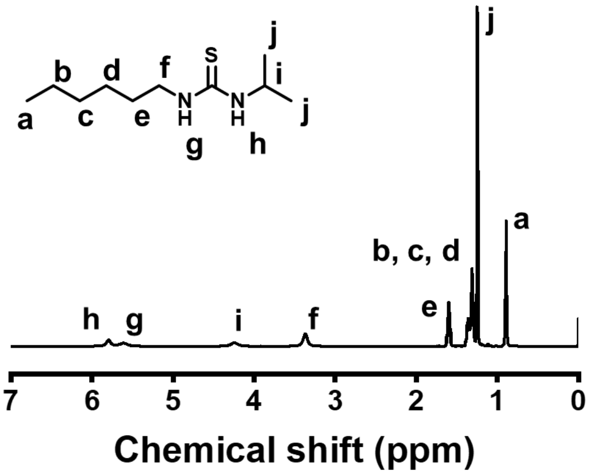

Supplementary Fig. 12 | ^{1}H-NMR spectrum of HITU.

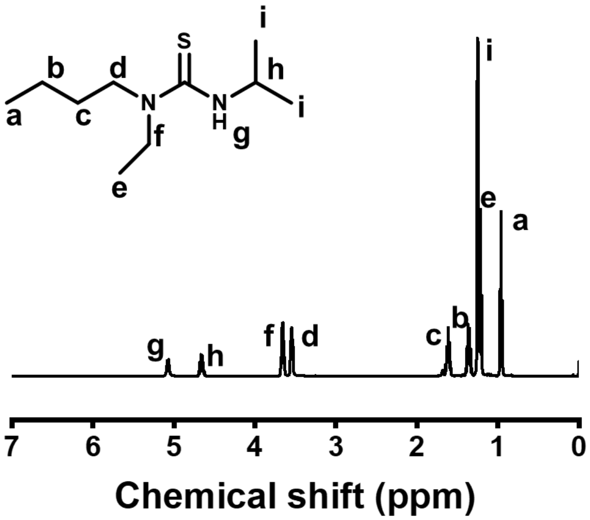

Supplementary Fig. 13 | ^{1}H-NMR spectrum of BEIT.

Supplementary References:

1. Miao, W. et al. Structural tuning of polycaprolactone based thermadapt shape memory polymer. *Polym. Chem* **11**, 1369-1374 (2020).
2. Sahu, S., Sahoo, P. R., Patel, S. & Mishra, B. K. Oxidation of thiourea and substituted thioureas: a review. *J. Sulphur Chem.* **32**, 171-197 (2011).
3. Stefaniu, C. et al. Rigid urea and self-healing thiourea ethanolamine monolayers. *Langmuir* **31**, 1296-1302 (2015).
